# The Prevalence of Phenylketonuria in Arab Countries, Turkey, and Iran: A Systematic Review

**DOI:** 10.1155/2018/7697210

**Published:** 2018-04-18

**Authors:** Ashraf El-Metwally, Lujane Yousef Al-Ahaidib, Alaa Ayman Sunqurah, Khaled Al-Surimi, Mowafa Househ, Ali Alshehri, Omar B. Da'ar, Hira Abdul Razzak, Ali Nasser AlOdaib

**Affiliations:** ^1^King Abdullah International Medical Research Center (KAIMRC) and College of Public Health and Health Informatics, King Saud Bin AbdulAziz University for Health Sciences, Riyadh, Saudi Arabia; ^2^Department of Epidemiology, School of Health Sciences, University of Tampere, Tampere, Finland; ^3^Newborn Screening and Biochemical Genetics Lab, Research Center, King Faisal Specialist Hospital & Research Center, Riyadh, Saudi Arabia; ^4^Ministry of Health and Prevention, Dubai, UAE; ^5^King Salman Center for Disability Research, Riyadh, Saudi Arabia

## Abstract

**Background/Objectives:**

This paper seeks to identify the prevalence of Phenylketonuria (PKU) in Arab countries, Turkey, and Iran. The study reviewed the existence of comprehensive national newborn screening programs and reported consanguinity rates.

**Methods:**

A computer based literature search was conducted using relevant keywords to retrieve studies conducted on PKU. A total of 34 articles were included. Prevalence was categorized based on the type of screening method used for PKU diagnoses.

**Results:**

The prevalence of classical PKU diagnosed through a comprehensive national newborn screening program ranged from 0.005% to 0.0167%. The highest prevalence was reported in Turkey at 0.0167%, whereas the lowest prevalence was reported in the UAE, 0.005%.

**Conclusion:**

The findings of this review emphasize the need for the establishment of more efficient reporting systems in these countries that would help measure Disability-Adjusted Life Year (DALY) in order to estimate the overall societal burden of PKU.

## 1. Introduction

Phenylketonuria (PKU) (OMIM 261600) is an autosomal recessive inborn error of phenylalanine (Phe) metabolism, occurring in approximately 1 : 15,000 people. PKU is mainly caused by a deficiency of phenylalanine hydroxylase (PAH; 612349), the enzyme that catalyzes the hydroxylation of phenylalanine to tyrosine [1]. Hyperphenylalaninemia can also be due to defects in the regeneration or biosynthesis of the enzyme cofactor tetrahydrobiopterin (BH4). If untreated or undiagnosed, the neurotoxic effects of excess phenylalanine can lead to impaired postnatal cognitive development. Both types of hyperphenylalaninemias (PAH and BH4 deficient) are thought to be heterogeneous disorders that vary from severe, for example, classical phenylketonuria (PKU), to mild, benign, and transient forms. Enzyme deficiency yields a spectrum of disorders such as mild hyperphenylalaninemia, mild phenylketonuria, and classic phenylketonuria. Classic phenylketonuria is a result of near complete or complete deficiency of phenylalanine hydroxylase activity which will lead to profound and irreversible intellectual disability in the absence of dietary restriction of phenylalanine. Mild hyperphenylalaninemia and mild phenylketonuria are linked to a lower risk of impaired cognitive development if left untreated [2]. All of the aforementioned are included in OMIM 261640 with cytogenetic location: 12q23.2. Deficiency of BH4 is a rare disorder that changes the levels of various substances in the body, including phenylalanine [3]. Clinical manifestations of BH4 deficiency include intellectual disability, neurological deterioration, difficulty swallowing, movement disorders, behavioral problems, seizures, and an incapability to control body temperature.

Today, the clinical manifestations of classic phenylketonuria are rarely reported in the developed countries, where newborn screening (NBS) is prevalent. NBS has permitted the early detection and successful treatment with diet low in phenylalanine. The first NBS program emerged in the United States in the early 1960s [4] and became universal in most developed countries [5]. With the use of state-of-the-art detection methods such as tandem mass spectrometry, PKU can be diagnosed readily in blood specimens collected by heel-prick from newborns, 24 hours of age, and spotted onto a filter paper that contains all their demographic information [6].

Newborn screening is the principal population-based public health screening program which is being practiced at present across the globe [7]. In case of PKU, it was found that the prevalence differs between different populations [8]. The incidence of PKU varies among ethnic groups and geographic regions worldwide [9]. For example, Caucasians are effected at a rate of 1 : 10,000 birth in the United States [10]. In Europe, the highest incidence has been observed in Ireland at a rate of 1 : 4,500 [11]. It is also common in few parts of China, while it is rarely observed in African nations. In Turkey, an incidence as high as 1 : 2,600 has been reported [12]. Supplementary information about incidence rates in different countries is mentioned in Table 1 [12–14]. Given its autosomal recessive inheritance, consanguinity among carrier couples is considered as the main risk factor for PKU [3].

This review aims to investigate the prevalence and incidence of PKU in Arab countries, Turkey, and Iran, which share similar culture and customs. This study will also explore the role of NBS programs in estimating PKU prevalence and incidence.

## 2. Method

Electronic search using Pub Med, Embase, and Google Scholar was conducted to extract articles addressing the epidemiology of Phenylketonuria in Arab countries, Turkey, and Iran. Key words used for our search included Phenylketonuria or PKU or aminoacidopathies or metabolic disorders or inborn error metabolism and (prevalence or incidence or frequency) and (newborn screening program or selective screening or national neonatal screening or tandem mass spectrometry) and (Saudi or Kuwait or Oman or United Arab Emirates or Bahrain or Qatar or Egypt or Iraq or Syria or Jordon or Sudan or Libya or Tunisia or Algeria or Morocco or Palestine or Lebanon or Yemen).

### 2.1. Inclusion and Exclusion Criteria

Inclusion criteria were English language articles published in peer-reviewed journals from January 1982 to December 2017, studies focusing on prevalence/incidence of PKU and NBS programs in the countries mentioned above. Any case reports/series or articles tackling treatment, molecular mutation, and molecular diagnosis were excluded.

### 2.2. Selection and Data Extraction

Comprehensive search terms such as Phenylketonuria or PKU or aminoacidopathies or metabolic disorders or inborn error metabolism were systematically applied along with Boolean operators. A broad search of Pubmed and Embase databases yielded 2487 records. After removing duplicate records, a total of 1772 were identified in our search, of which, 1702 were irrelevant and were excluded based on title/abstract screening. Finally, 70 full-text articles were assessed for eligibility and were screened against the inclusion criteria (including 18 Arab countries). A total of 48 articles were further eliminated because PKU prevalence/incidence was neither mentioned nor were the researchers able to extract data or self-calculate prevalence. A secondary search was performed by cross-referencing and using the same keywords in Google Scholar that resulted in a total of (12) articles, which did not appear in our original PubMed and/or Embase search. Consequently, the total number of articles included in this systematic review was 34. The review was conducted by two professionals in the field of epidemiology and public health. Any disagreements between the two researchers were solved by consensus.

## 3. Results

A total of 34 prevalence/incidence studies conducted in different years and regions were included in this review (see Table 2). In some of the prevalence/incidence studies, prevalence was self-calculated in 9 articles and corrected in 11 articles. Prevalence/incidence studies were further categorized to the type of study whether it was a national NBS program (*n* = 5 articles) [6, 15–18], regional/governorate NBS program (*n* = 6 articles) [19–24], selective screening for newborns (*n* = 6 articles) [25–30], selective screening of sick/symptomatic newborns and/or infants, children, and adults (*n* = 9 articles) [31–39], selective screening for both newborns and sick/symptomatic newborns and/or infants, children, and adults (*n* = 4 articles) [40–43], or selective screening for sick/symptomatic children and adults from institutions for mentally challenged (*n* = 3 articles) [44–46]. In addition, a study conducted in Turkey addressed PKU prevalence among newborns, sick/symptomatic subjects, and mentally challenged individuals. For all selected studies, prevalence of classical PKU, BH4 dependent PKU, and mild–moderate HPA were calculated as a percentage, and as a rate per 100,000 neonates/population, also presented in Table 2 [43]. Furthermore, consanguinity rate is indicated wherever available.

To conduct a reliable comparison of PKU prevalence, and as most of the studies reported prevalence and/or incidence in different ways, we first sought to unify the prevalence calculation in the form of percentage and rate per 100,000 of the screened population. Then, we categorized the studies by the population used to estimate prevalence into either national, regional, and selective screening programs or studies conducted in institutions for the mentally challenged. Moreover, a comparison was conducted using classical PKU prevalence as the most severe form in addition to the fact that not all (only few studies) gave estimates for BH4 dependent PKU (6 studies) and mild/moderate HPA (13 studies). Prevalence of self- calculation for PKU (including classical type or BH4 dependent PKU and HPA) was generated by extraction of the available information from articles included in this study by dividing the number of cases by the number of life births or sample size in the study during a specific year. Prevalence calculations were tabulated and expressed as percentage or as rate per 100,000 of population screened. Though we calculated PKU prevalence for all studies considered, only national programs will yield solid estimates.

Prevalence of classical PKU extracted or self-calculated from articles using comprehensive national NBS programs ranged between 0.005% and 0.0167%. The corresponding range for regional/governorate NBS programs was 0.0015% to 0.0213%. Selective screening programs of newborns gave prevalence of 0.0072% to 0.0381%. However, in articles estimating PKU via selective screening of sick/symptomatic subjects [newborns, infants, children, and adults], the prevalence was reported to be between 0.0273% and 11.1%. Prevalence in institutions caring for mentally challenged individuals ranged from 1.55% to 4.722%. Four articles reported prevalence based on selective screening of both apparently healthy newborns and sick/symptomatic newborns (i.e., neonates who have missed newborn screening, thus, becoming symptomatic or acting abnormally in any way). Prevalence for selectively screened newborns was 0.0198%–0.0250% and prevalence for sick/symptomatic subjects ranged from 1.917% to 2.974%.

## 4. Discussion

The review addressed and sought to shed light on the epidemic of PKU in the Arab countries, Turkey, and Iran. To our knowledge, this is the first systematic review conducted to summarize the prevalence of PKU in these countries. Despite the lack of published data on PKU prevalence in many Arab countries such as Algeria, Syria, Libya, Sudan, and Yemen, most likely due to the absence or limitation of comprehensive screening programs [47], our review still reflects the high prevalence of PKU in Saudi Arabia, United Arab Emirates (UAE), Turkey, Gaza Strip, Sulaimani, the Baghdad region in Iraq, and the Fars region in Iran. Our results show that prevalence of classical PKU in countries having national newborn screening programs ranges from 0.005% to 0.0167%. The highest prevalence was reported for Turkey in 1995 (0.0167%) [18] and the lowest one for the UAE in 2003 (0.005%) [15]. In regions conducting NBS, prevalence ranged from 0.0015% in the Mazandaran Province [24] to 0.02% in the Fars region in south-central Iran [22].

The prevalence of classical PKU among selective NBS studies ranged between 0.0072% and 0.038%. The lowest prevalence was reported for the Aramco Province in Eastern Saudi Arabia (0.0072%) [25] and the highest for Ankara (0.038%) [27]. Other studies estimated prevalence through selective screening for sick/symptomatic newborns and/or infants, children, and adults such as the ones conducted in Bahrain, Kuwait, Oman, Egypt, Jordan, Lebanon, and Iran. PKU prevalence among sick/symptomatic newborns was highest in the Jordan study (8%) [35] (due to relatively a small sample size) and the lowest in the study was conducted by Golbahar et al. with 0.0273% [31]. Among mentally challenged individuals, the highest prevalence was noted in Turkey during 1990 (4.722%) [43] and the lowest in Kuwait (1.55%) [44]. Prevalence among sick/symptomatic subjects was the highest in Egypt during 2009 (2.5%) and the lowest in Turkey in 1990 (0.02%).

To date, only a few countries such as Saudi Arabia, UAE, Qatar, and Turkey in the region have implemented comprehensive national NBS programs with relatively high coverage that aim for early detection of PKU along with other treatable disorders in an attempt to reduce disability rates. The percentage uptake (or coverage) of newborn screening in the UAE was increased from 50% in 1998 to reach 95% in 2010, with a prompt increase in the year 2003 [16]; however, these levels are still below the international coverage standards (99%) [48].

Unfortunately, our search failed to find any published data showing the prevalence of PKU in Qataris. The prevalence of PKU in Saudi Arabia was 0.0068%. In UAE, PKU prevalence was 0.0081% analogous to the prevalence (~0.0073%) for the Aramco Province in Eastern Saudi Arabia. Consecutive studies on PKU prevalence in UAE have demonstrated an increase in prevalence with time from 0.005% in 2003 to 0.0068 in 2014, and finally 0.008% in 2016.

A global comparison of incidence rates between countries with nationwide NBS programs shows that Japan, among Asian countries, reports the lowest rate with 1 : 125,000, whereas incidence in China is 1 : 17,000 [10]. Saudi Arabia is close to the PKU incidence of Japan at 1 : 14,623 [6] and UAE 1 : 12,369 [17]. On the other hand, the incidence rate among Caucasians in North America (1 : 10,000) is lower than those reported for Japan and China [49]. A similar study give reported incidence for Australia [10]. Among European countries, incidence rates among Saudis are higher than the rate of 1 : 4,500 reported for Ireland [11] but comparable to rates recorded in Denmark 1 : 12,000, France 1 : 13,500, Norway 1 : 14,500, and finally UK 1 : 14,300 [10].

The consanguinity rate is very high in Arab countries as reported by most of the articles in our search. For example, 9 out of 11 PKU patients in Oman [33] and 8 out of 9 PKU patients in Kuwait had consanguineous parents [32]. Other studies conducted in Iraq [37] indicated that all 7 cases detected with PKU had consanguineous parents. These findings were consistent with previous studies where 57% of PKU patients in Egypt [26], 60% of PKU cases in Gaza Strip [19], 86.6% of PKU patients in Iran [22], and 34 patients out of 43 PKU cases from Iran [31] had consanguineous parents. Similarly, a recent study conducted in 2017 in Mazandaran Province in Iran indicated that parental family relationships among confirmed PKU cases were 53.6% [24]. Congruently, another study from Iraq, Sulaimani city [20], reported only one case diagnosed with PKU being a product of consanguineous parents.

Addressing some recent articles, consanguinity rates among all cases with different metabolic disorder including PKU were reported. For example, a Jordanian study [35] reveals that out of 151 families, 137 cases had parental consanguinity. Similarly, Al-Jasmi et al. [17] in UAE declared that, among all metabolic disorders detected including PKU, consanguinity was 81.5%. Alternatively, Selim et al. [34] showed that 88% of patients were born to consanguineous parents in Egypt. These results concur with Moammar et al. [25] findings in Saudi Arabia revealing all detected cases to have consanguineous parents. A study conducted in Gaza in contrast confirmed that some PKU cases were not consanguineous [19]. Nevertheless, it fails to mask the fact that most of the studies reporting the cases arose from consanguineous marriages.

## 5. Limitations

There were certain limitations to this review. First, our search was limited to publications in English. However, most if not all research conducted in the Arab world is published in English. One major drawback was attributed to the study design itself, in particular for prevalence/incidence studies where data from most of the articles were based on retrospective data collection either from medical records or registries. This kind of routine data has its own disadvantages such as incompleteness or inaccurateness. Other limitations include small sample size (63 samples from sick/symptomatic children in Iraq) in Rabab Thijeel study [37]. There is still an ambiguity with regard to the high prevalence of PKU (11.1%) that cannot be generalized. Likewise, another possible limitation involved the way prevalence/incidence calculations were reported in some studies, where some articles used the denominator as a number of all live births during the study period and not the actual number of screened subjects. Others perform PKU estimations by using a total number of abnormal cases as a denominator instead of total number screened. For those incorrect estimates, corrections were made and documented in Table 2.

## 6. Implications for Future Research

PKU if not detected and treated early will lead to disability which presents a great socioeconomic burden for any country. Unfortunately, only few countries in the region including Saudi Arabia, UAE, Qatar, and Turkey have established active and comprehensive national NBS programs for PKU along with other disorders. More studies are needed in the region to monitor and study PKU. At the public level, and since consanguinity is the main factor of having the disorder in our region, continuous awareness campaigns through media, schools, and universities are recommended to educate the public about potential health risks posed by marriage between close relatives. Genetic counselors also play a big role in educating and helping the parents and affected siblings in not having another affected child during future pregnancy by introducing them to primary prevention such as prenatal diagnosis or Preimplantation Genetic Diagnosis (PGD). Issuing a policy through governments to mandate the screening test for every newborn is one effective approach to reduce PKU. Due to the rarity of specialized experts in this field, physicians, scientist, lab technologist, and governments should support training programs to compensate for this inadequacy.

## 7. Conclusion

In light of this review, our search demonstrated the need for establishment of more research work so as to investigate the true prevalence of PKU in our region using comprehensive population screening tests. The data in regard to prevalence, follow-up, and identification of other possible risk factors or other disease spectrum associated with PKU is scarce in our region. Our research through PubMed, Embase, and Google Scholar failed to find published data about reliable or recent PKU prevalence in many Arab countries such as Syria, Yemen, Libya, Morocco, Algeria, Tunisia, and Sudan.

Future research should also focus on measuring the Disability-Adjusted Life Year (DALY) to demonstrate overall burden of this disease as well as other genetic diseases. Estimating DALY is another successful measure to estimate years of life lost due to premature mortality (YLL) and years of life lived with disability (YLD). Providing such data will definitely give true estimates of this problem and allow for effective intervention programs to reduce disease burden.

## Figures and Tables

**Table 1:** Incidence of PKU by populations. Source: [11, 13, 14].

Regions	Countries	Incidence of PKU
Asian populations	China	1 : 17,000
	Korea	1 in 41,000
	Japan	1 in 125,000

European populations	Ireland	1 in 4,500
	Scotland	1 in 5,300
	Czechoslovakia	1 in 7,000
	Hungary	1 in 11,000
	Denmark	1 in 12,000
	France	1 in 13,500
	Norway	1 in 14,500
	United Kingdom	1 in 14,300
	Italy	1 in 17,000
	Finland	1 in 200,000

North America	United States (Caucasians)	1 in 10,000
	Canada	1 in 22,000

Oceania	Australia	1 in 10,000

**Table 2:** Illustration categorization of the studies based on the type of screening used. Prevalence as % and rate per 100.000 neonates and or sick/symptomatic subjects were computed. NA: information is not available; ^*∗*^prevalence among citizen only, ^a^self-calculated prevalence, and ^c^corrected information. Remarks column indicates the way of prevalence/incidence estimated by articles if different from computed and states any self-computing prevalence and correction made in this review.

Type	Study and country setting	Age at sampling	Sample size	Prevalence									Consanguinity	Remarks
				Classical PKU			Biopterin defect (BH_4_)			HPA (mild and moderate)				
				Number of cases	(%)	Per 100,000 neonates/sick	Number of cases	(%)	Per 100,000 neonates/sick	Number of cases	(%)	Per 100,000 neonates/sick		
National NBS program	[6] Saudi Arabia; 2017, King Faisal Specialist Hospital and Research Center, King Salman Center for Disability Research, King Saud bin Abdulaziz for health Science, King Abdulaziz Medical City, Ministry of National Guard-Health Affairs, King Fahad Medical City, Children Hospital, Armed Forces Medical Service Directorate, Security Forces Hospital	After 24 hr. of birth	775000	53	0.0068	6.84	NA	—	—	NA	—	—	No information	Incidence Rate reported in article as 1 : 14245 *and should be corrected to 1 : 14623*
	[15] United Arab of Emirates, 2000, Ministry of Health, National screening center, Tawam Hospital	5th day for discharged newborns and before discharge for those admitted for >5 days	138718	7	0.0050	5.05	NA	—	—	NA	—	—	No information	Incidence Rate reported in article as 1 : 20050 and should be corrected to 1 : 19816.9
	[16] United Arab of Emirates, 2014, Ministry of Health, National screening center, Tawam Hospital	3rd day after birth (≥48 hr) and before discharge for those admitted for >3 days	750365	51	0.0068	6.80	1.00	0.00013	0.13	NA	—	—	No information	PKU incidence rate reported in article as 1 : 14544 and should be corrected to 1 : 14713 not including BH_4_ defect and to 1 : 14430.1 if including BH_4_ dependent PKU case
	[17] ^*∗*^United Arab of Emirates, 2016^a,c^, United Arab Emirates University, Al-Ain, Tawam Hospital	48 hr. of age and before discharge for those admitted for >3 days	136049	11	0.0080	8.09	NA	—	—	NA	—	—	Among PKU not indicated. But among all 55 metabolic cases detected inclusive PKU consanguinity was 81.5%	Self-calculated prevalence among citizens. Article estimates overall incidence of metabolic disorders included in program. Correction of some of all Emirates live birth in Table 1 (2011–2014) to be 136058 instead of 136049
	[18] Turkey, 1995^c^, Hacettepe University, Departments of Nutrition and Metabolism, Molecular biology, Dietetics and Neonatology, Ankara	Samples collected before discharge & test repeated if collected <24 h	576122	96	0.0166	16.66	1.00	0.00017	0.17	46.0	0.008	7.98	45.7% of marriages among PKU families were consanguineous. 30.9% were first degree relative marriages, 5.6% second degree, 7.2 others and 54.3 were nonconsanguineous	Incidence of classical PKU in article table reported as 1 : 6000. For persistent HPA as 1 : 12500. The total of both as 1 : 4500 that should be corrected to 1 : 4057
Regional/governorate newborn screening program	[19] Palestine Gaza Strip, 2015^c^, Biology Department, Al-Azhar University, College of Public health, Gaza Central Laboratories, Ministry of Health	Average age 13.5 days	1022207	65	0.0063	6.36	NA	—	—	NA	—	—	60% of PKU parents were first cousins, while 7.7% with no consanguinity	Correction: article used the total population live birth in calculating prevalence. However, it should be calculated using total number of newborn screened in 2000 which is mentioned as 13175 and including the 11 cases that had been excluded due to death. So, corrected prevalence should be (13175/76)*∗*100 = 0.576% or ~577 : 100000
	[20] Iraq, Sulaimani City, 2015, Iraq, Department of Pediatrics, School of Medicine, University of Sulaimani and Sulaimani Pediatric Teaching Hospital	3–10 days	8255	1	0.0121	12.11	NA	—	—	NA	—	—	The parents of this case were consanguineous	Incidence in article reported as 1.2 : 10000 neonates
	[21] Iraq, Baghdad/Al-Karkh Directorate, 2016^a^, Alkindy College of Medicine, University of Baghdad, AlKarkh Health Directorate, Ministry of Health	Within 3–5 days up to 2 months	80409	6	0.0075	7.46	NA	—	—	NA	—	—	No information	Self-calculated Prevalence. Article did not report prevalence of PKU. Data extracted from table & figure then calculated
	[22] Iran, Fars province, Shiraz, 2009, Human Genetics Research Group, Iranian Academic Center for Education, Culture and Research, Paramedical School of Shiraz University of Medical Science, Iran Center of Blood Transfusion of Shiraz, Department of Mathematics, Yasuj University	72 hours after birth	70477	15	0.0213	21.28	NA	—	—	NA	—	—	The frequency of familial marriages in these children parents were 86.6%	Incidence rate reported in article as 1 : 4698
	[23] Iran, Fars province, 2010, Pediatric Endocrinologist, PKU Center, Naderkazemi Clinic, Department of Pediatrics, Department of Medical Technology, Paramedical School, Neonatal Screening Laboratory in Shiraz University of Medical Sciences	3–5 days after birth	175235	28	0.0160	15.98	NA	—	—	1.0	0.001	0.57	No information	Reported incidence of PKU was 1.6 : 10000. It also indicate incidence of malignant PKU to be 3 : 100. Two patients had transient HPA.
	[24] Iran, Mazandaran Province, Department of Pediatrics, School of Medicine, Clinical Research Development Unit of Bou Ali-Sina Hospital, Diabetes Research Center, Research Development Unit of Referral Laboratory, Deputy of Health, Deputy of Health Management, Department of Pharmacology, all from Mazandaran University of Medical Science. Deputy of Health, Babol University of Medical Science	During the days 3–5 after birth	407244	6	0.00147	1.47	NA	—	—	21	0.005	5.16	Parental relationship observed in 16 cases (53.6%)	PKU incidence was reported to be 0.66 in 10,000, while nonclassic PKU cases were not detected
PKU selective screening for newborns	[25] Saudi Arabia, Aramco, 2010, Saudi Aramco Medical Facilities in the Eastern Province	Not indicated	165530	12	0.0072	7.25	4.00	0.0024	2.41	NA	—	—	Almost all of detected cases were consanguineous	Incidence Rate reported in article for classical PKU is 7 : 100000 live birth and for BH_4_ defect is 2 : 100000
	[26] Egypt, Menofiya, 2009, Pediatrics Department, National Liver Institute, Menoufiya University, Biomedical Genetics Department, National Research Center	Mean age 9.3 ± 2.43 days	3000	1	0.0333	33.33	NA	—	—	NA	—	—	Positive consanguinity was found in 57% of the samples	Incidence Rate reported in article as 1 : 3000 (0.03%)
	[27] Turkey, 1986, Department of Metabolism and Department of Neonatology, Institute of Child Health, Hacettepe University, General Maternity Hospital, Ankara	Samples collected before discharge & test repeated if collected <24 h	20979	8	0.0381	38.13	NA	—	—	4.0	0.019	19.07	No information	Article reported Incidence of typical PKU as 1 : 2622 and that for HPA as 1 : 5243 and the overall is 1 : 1747
	[28] Lebanon, 2003^a^, Department of Pathology and Laboratory Medicine and Pediatrics, American University of Beirut	2-3 days	9117	1	0.0110	10.97	NA	—	—	1.0	0.011	10.97	No information	Self-calculated Prevalence. Article did not state prevalence of PKU. Data extracted from table and calculated for both classical PKU and HPA.
	[29] Lebanon, 2015, Medical genetics Unit in Saint –Joseph University, Epidemiology and Population Studies Department and Department Of Pediatrics and Adolescent Medicine at the American University of Beirut, Faculty of Science at the Lebanese University	Not indicated	126000	18	0.0143	14.29	7.00	0.0055	5.55	NA	—	—	No information	
	[30] Iran, 1982^c^, Human Genetic and Anthropology Unit, Department of Human Ecology, School of Public Health and Department of Pediatrics, Medical School, University of Tehran	4–8 days	8633	1	0.0116	11.58	NA	—	—	7.0	0.081	81.08	No information	Reported incidence was 1 : 8000, should be corrected to 1 : 8633. Mild HPA cases normalized after retesting
Selective screening of sick newborns or/and infants, children and adults	[31] Bahrain, 2013, Department of Molecular Medicine, Colleges of Medicine and Medical Science, AGU, Manama	3–90 days	1986	3	0.1511	151.06	NA	—	—	NA	—	—	21 out of 25 diagnosed patients had consanguineous parents	Article stated detection rate as 1 : 662 (incidence among screened) and incidence rate among all live birth during this period as 1 : 22188
	[32] Kuwait, 1988, Department of Clinical Biochemistry, Al-Sabah Hospital	13% neonates, 26% infants (<1 year of age), 43% older children and 18% adults (>12 year)	800	9	1.1250	1125.00	NA	—	—	1.0	0.125	125.0	Out of 9 patients, 8 had consanguineous parent and one nonconsanguineous	Benign HPA only one case. Author only included 9 PKU in prevalence calculation
	[33] Oman, 2012^a^, Department of Biochemistry, Department of Child Health, Sultan Qaboos University	No age	1100	8	0.7273	727.27	3.00	0.2727	272.72	NA	—	—	9 out of 11 PKU patients had consanguineous parents	Self-calculated Prevalence. Article did not report prevalence of PKU. Data extracted from table and calculated for both classical and BH_4_ dependent PKU
	[34] Egypt, Cairo, 2014^c^, Department of Pediatric Neurology, Department of Pediatric Genetics and Department of Clinical and Chemical Pathology in Cairo University. Inherited Metabolic Disease Unit, Cairo University Children Hospital	2.5 months to 6.6 years	3380	100	2.9586	2958.58	NA	—	—	NA	—	—	Out of 203 different metabolic disorder detected, 178 of patients were born to consanguineous parent's ~ 88%	Article stated prevalence of PKU from total abnormal 203 cases detected (100/203)*∗*100 = 49.3%. Corrected prevalence should be (100/3380)*∗*100 = 2.96%
	[35] Jordan, 2012, Department of Pediatrics, Metabolic Genetics Clinic, Queen Rania Al-Abdullah Children Hospital, King Hussein Medical Center	One to 50 months	212	17	8.0189	8018.87	NA	—	—	NA	—	—	137 out of 151 families having different metabolic disorder showed parental consanguinity	
	[36] Lebanon, 2013^a,c^, Department of Pediatrics and Adolescent Medicine and Department of Pathology and Laboratory Medicine at the American University of Beirut Medical Center	2 months to 21 years	2921	90	3.0811	3081.1	NA	—	—	NA	—	—	Included other metabolic disorders were 60% and parents were first cousins in 35%	Article stated prevalence of PKU from total abnormal 203 cases detected (90/112)*∗*100 = 42.7%. Corrected prevalence should be (90/2921)*∗*100 = 3.08%. Author mentioned that during last three years of the study, 49750 newborns were screened out of which 13 cases of PKU were detected without reporting prevalence. So self-calculated Prevalence of PKU from this information is 0.026%
	[37] Iraq, 2013, Department of Pediatrics, College of Medicine, Baghdad University and Children Welfare Teaching Hospital Medical City Complex	>1 year and <5 years	63	7	11.111	11111.1	NA	—	—	NA	—	—	All PKU cases were related to consanguineous marriages	
	[38] Iraq, 2016, Child Welfare Teaching Hospital and Al-Emamain Al Kadhemyian Teaching Hospital, Baghdad	9.3% neonates (0–30 days of age), 34% (>1–5 years), 9.3% >5 years.	1758	19	1.0807	1080.8	NA	—	—	1.0	0.057	56.88	Out of 1758 sick patients, 174 cases had consanguineous parents (9.8%)	
	[39] Iran, Shiraz, 2002^a,c^, Department of Biochemistry, Department of Pediatric, Shiraz University of Medical Sciences	Not indicated	106151	29	0.0273	27.3	NA	—	—	10.0	0.009	9.42	34 patients with PKU out of the 43 had consanguineous parents	Reported incidence of PKU in article after Jan 1996 is 1 : 3672. However, it was mentioned that total number of PKU cases detected out of 1044 patient selectively screened was 43 cases. 33 classical PKU and 10 milder cases. So corrected calculated prevalence of classical PKU among selectively screened is (33/1044)*∗*100 = 3.16% or 3161 : 100000
Selective screening for both newborns and sick newborns and/or infants, children and adults	[40] Kuwait, 2007^a^, Department of Pharmaceutical Chemistry and Pharmacy Practice, Faculty of Pharmacy at Kuwait University and Department of Pediatrics at Sabah Hospital													
	Among newborns Among sick	Not indicated	1520 362	0 1	0.000 0.2762	0.00 276.2	NA NA	— —	— —	3.0 0.0	0.197 0.0	197.37 0.00	No information	Self-calculated Prevalence among sick subjects. Article did not report prevalence of PKU. Data extracted from table and calculated. For all those screened (sick or newborns). Overall classical PKU prevalence can be estimated as (1/1520)*∗*100 = 0.066%
	[41] Egypt, 2009^a^, Clinical and Chemical Pathology and Pediatrics Departments, Faculty of Medicine, Cairo University and Ministry of Health and Population													
	Among neonates Among sick/symptomatic	3–7 days 3 months to 15 years	16000 550	4 14	0.0250 2.5455	25.0 2545.4	NA	—	—	—	—	—	No information	Self-calculated prevalence among sick subjects. Article report incidence of PKU among newborns as 1 : 4000. For all those screened (sick & newborns), overall classical PKU prevalence can be estimated as (18/16550)*∗*100 = 0.11%
	[42] Egypt, 2016^c^, Clinical and Chemical Pathology and Department of Pediatrics in Cairo University, Inherited Metabolic Disease Unit, Cairo University Children Hospital													
	Among neonates Among sick/symptomatic	3–7 days 1 week to 15 years	25276 3900	5 116	0.0198 2.9744	19.8 2974.3	NA	—	—	NA	—	—	No information	Article stated that PKU cases among newborns are 1 : 5000. Also, estimated birth prevalence among newborns and sick subjects as percent from number of abnormal cases not total screened as 38.5% and 49.3%, respectively. Corrected estimates should be done using total number screened as calculated in table
	[43] Turkey, Ankara, 1990^c^, Institute of Child Health, Department of Metabolism, Hacettepe University, Ankara, Department of Pediatrics, Free University of Berlin													
	Among Selected high risk infants Among healthy newborn	Not indicated	6050 170466	116 39	1.9174 0.0229	1917.4 22.9	NA NA	— —	— —	NA 19.0	— 0.011	— 11.15	In infant's selective screening, there was parental consanguinity in 72% of the all 225 detected cases	Reported incidence of typical PKU among newborns as 1 : 4370, persistent HPA as 1 : 8971 and overall incidence as 1 : 2874. The total should be corrected to 1 : 2939
Selective screening for sick children and adults from mental retardation institutes	[43] Turkey, Ankara, 1990, Institute of Child Health, Department of Metabolism, Hacettepe University, Ankara, Department of Pediatrics, Free University of Berlin	Not indicated	10800	510	4.7222	4722.2	NA	—	—	NA	—	—	Among mentally retarded children, 45% of cases had parental consanguinity and the rest were not	
	[44] Kuwait, 1987, Kuwait Medical Genetics Center, Maternity Hospital	5–45 Years	451	7	1.5521	1552.1	NA	—	—	NA	—	—	Parental consanguinity in all 7 patients. In six cases parents were first cousins and in one case distant relatives but from the same tribe	
	[45] Iran, Isfahan, 2003^a^, Department of Biology, Genetics division at Isfahan University	Not indicated	611	26	4.2553	4255.3	NA	—	—	8.0	1.3	130.9	68% of the cases, parents were first cousins	Article stated the prevalence of all HPA as (34/611)*∗*100 = 5.56%. Also estimated frequency of all HPA among all mentally retarded individuals in the institute as (34/1541)*∗*100 = 2.2%. So, the prevalence of true PKU cases among screened 611 subject was self-calculated to be 4.25%
	[46] Iran, Tehran, 2009, Department of Biology, Grand Vally State University, Genetic Center, Beheshti University of Medical Science in Tehran, Iran, Department of Epidemiology, School of Health and Nutrition, Shiraz University of Medical Science, Student Research Center, Tehran University of Medical Science, Department of statistics, Grand Vally state University Allendale, Mi, USA, Tehran Province Welfare and Rehabilitation Organization	Average age 13.5 days	4963	104	2.0955	2095.5	NA	—	—	21.0	0.423	423.13	No information	Article reported prevalence of classical PKU among all mentally retarded individuals in Iran as 2.1% and prevalence of mild HPA as 0.44%. The prevalence among inmates sheltered in Tehran only was 2.81% and other cities were 1.68%
